# Book Reviews

**Published:** 1910-05

**Authors:** 


### BOOK REVIEWS

ESSENTIALS OF HISTOLOGY. By Louis LeRoy, B. S., M. D., Prof. of Theory and Practice of Medicine, College of Physicians and Surgeons, Memphis, Tenn., Etc. Arranged with questions following each chapter; 114 illustrations; 4th edition, revised and enlarged. W. B. Saunders & Co., Philadelphia.

Leroy has collected the essentials of histology in a limited space and presented the subject in a clear, attractive manner.

The usefulness of Saunders' Question-Compends is attested by their phenomenal sale, and we need but say that this present revised volume is up to the usual high standard.

ANATOMY AND PHYSIOLOGY FOR NURSES. By LeRoy Lewis, M. D., Surgeon to and Lecturer on Anatomy and Physiology for Nurses at the Lewis Hospital, Bay City, Michigan. Second Revised Edition. 12 mo. of 344 pages, with 161 illustrations. Philadelphia and London: W. B. Saunders Company, 1910. Cloth, $1.75 net.

While this book was intended for nurses, it really affords the medical student a most useful book from which to acquire a working knowledge of these subjects. In fact, it is the reviewer's opinion that some day in the future, anatomy, histology and such subjects which are so quickly forgotten by us all, will not receive so much time during the first two years of college, but we will be content to teach them well about what is found in this work and devote a much larger time to beside teaching and bringing the student directly in contact with the diseased body, and thus accustom him to make better examinations and fewer theoretical guesses. For the nurse, Lewis' book is well worthy of praise.

SURGICAL DIAGNOSIS. By Daniel N. Eisendrath, M. D., Professor of Surgery in the Medical Department of the University of Illinois (College of Physicians and Surgeons). Second Revised Edition. Octavo of 885 pages, with 574 original illustrations, 25 in colors. Philadelphia and London: W. B. Saunders Company, 1909. Cloth, $6.50 net; Half Morocco, $8.00 net.

That diagnosis is of utmost importance in the treatment of disease, whether medical or surgical, all will admit; but in spite of its importance, it is often neglected on account of difficulties necessarily involved in certain conditions. It is to lessen these difficulties that Eisendrath has prepared this excellent treatise, the second edition of which we are now reviewing and find to be well up-to-date and thoroughly practical, having been approached chiefly from the clinical standpoint. The work is beautifully illustrated and almost equals a clinic.

